# Neighborhood Food Environment, Diet, and Obesity Among Los Angeles County Adults, 2011

**DOI:** 10.5888/pcd12.150078

**Published:** 2015-09-03

**Authors:** Nelly Mejia, Amy S. Lightstone, Ricardo Basurto-Davila, Douglas M. Morales, Roland Sturm

**Affiliations:** Author Affiliations: Amy S. Lightstone, Ricardo Basurto-Davila, Douglas M. Morales, Los Angeles County Public Health, Los Angeles, California; Roland Sturm, RAND Corporation, Santa Monica, California.

## Abstract

**Introduction:**

The objective of this study was to examine whether an association exists between the number and type of food outlets in a neighborhood and dietary intake and body mass index (BMI) among adults in Los Angeles County. We also assessed whether this association depends on the geographic size of the food environment.

**Methods:**

We analyzed data from the 2011 Los Angeles County Health Survey. We created buffers (from 0.25 to 3.0 miles in radius) centered in respondents’ residential addresses and counted the number of food outlets by type in each buffer. Dependent variables were weekly intake of fruits and vegetables, sugar-sweetened beverages, and fast food; BMI; and being overweight (BMI ≥25.0 kg/m^2^) or obese (BMI ≥30.0 kg/m^2^). Explanatory variables were the number of outlets classified as fast-food outlets, convenience stores, small food stores, grocery stores, and supermarkets. Regressions were estimated for all sets of explanatory variables and buffer size combinations (150 total effects).

**Results:**

Only 2 of 150 effects were significant after being adjusted for multiple comparisons. The number of fast-food restaurants in nonwalkable areas (in a 3.0-mile radius) was positively associated with fast-food consumption, and the number of convenience stores in a walkable distance (in a 0.25-mile radius) was negatively associated with obesity.

**Discussion:**

Little evidence was found for associations between proximity of respondents’ homes to food outlets and dietary intake or BMI among adults in Los Angeles County. A possible explanation for the null finding is that shopping patterns are weakly related to neighborhoods in Los Angeles County because of motorized transportation.

## MEDSCAPE CME

Medscape, LLC is pleased to provide online continuing medical education (CME) for this journal article, allowing clinicians the opportunity to earn CME credit.

This activity has been planned and implemented in accordance with the Essential Areas and policies of the Accreditation Council for Continuing Medical Education through the joint sponsorship of Medscape, LLC and *Preventing Chronic Disease*. Medscape, LLC is accredited by the ACCME to provide continuing medical education for physicians.

Medscape, LLC designates this Journal-based CME activity for a maximum of 1 ***AMA PRA Category 1 Credit(s)™***. Physicians should claim only the credit commensurate with the extent of their participation in the activity.

All other clinicians completing this activity will be issued a certificate of participation. To participate in this journal CME activity: (1) review the learning objectives and author disclosures; (2) study the education content; (3) take the post-test with a 75% minimum passing score and complete the evaluation at www.medscape.org/journal/pcd; (4) view/print certificate.

**Release date: September 3, 2015; Expiration date: September 3, 2016**

### Learning Objectives

Upon completion of this activity, participants will be able to:

1. Distinguish dietary habits among adults in a large US metropolitan area.
2. Assess the distribution of food outlets in a large US metropolitan area.
3. Evaluate the effect of food outlet distribution on the diet of adults.
4. Evaluate the effect of food outlet distribution on the body mass index of adults.

**EDITOR**

Camille Martin, Editor, *Preventing Chronic Disease*. Disclosure: Camille Martin has disclosed no relevant financial relationships.

**CME AUTHOR**

Charles P. Vega, MD, Clinical Professor of Family Medicine, University of California, Irvine

Disclosure: Charles P. Vega, MD, has disclosed the following relevant financial relationships: Served as an advisor or consultant for: Lundbeck, Inc; McNeil Pharmaceuticals; Takeda Pharmaceuticals North America, Inc.

**AUTHORS**

Nelly Mejia, PhD Candidate, MPhil, and Roland Sturm, PhD, RAND Corporation, Santa Monica, CA; Amy S. Lightstone, MPH, MA, Ricardo Basurto-Davila, PhD, MSc, and Douglas M. Morales, MPH, Los Angeles County Department of Public Health, Los Angeles, CA

DISCLOSURES: Nelly Mejia, PhD Candidate, MPhil, and Roland Sturm, PhD, RAND Corporation, Santa Monica, CA; Amy S. Lightstone, MPH, MA, Ricardo Basurto-Davila, PhD, MSc, and Douglas M. Morales, MPH, Los Angeles County Department of Public Health, Los Angeles, CA, have disclosed no relevant financial relationships.

Nelly Mejia, Roland Sturm, Amy S. Lightstone, Ricardo Basurto-Davila, and Douglas M. Morales do not intend to discuss off-label uses of drugs, mechanical devices, biologics, or diagnostics approved by the FDA for use in the United States. Nelly Mejia, Roland Sturm, Amy S. Lightstone, Ricardo Basurto-Davila, and Douglas M. Morales do not intend to discuss investigational drugs, mechanical devices, biologics, or diagnostics not approved by the FDA for use in the United States.

Ricardo Basurto-Davila does intend to discuss off-label uses of drugs, mechanical devices, biologics, or diagnostics approved by the FDA for use in the United States. Ricardo Basurto-Davila does intend to discuss investigational drugs, mechanical devices, biologics, or diagnostics *not approved* by the FDA for use in the United States.

## Introduction

Food environments have become an important topic in policy debates to stem the obesity epidemic (1–3). Overweight and obesity clusters in areas indicate a role of shared environments, including the possibility that there are “obesogenic” environments (4). A recurring theme is the role of the food environment, in particular the notion of “food deserts,” defined as lack of access to healthy affordable foods and that are more common in disadvantaged neighborhoods (5–7).

The evidence on how neighborhood food environment affects an individual’s diet and body mass index (BMI) continues to develop, but it remains tentative, more so than is presented in the media and policy arguments (8,9). Prominent hypotheses include that fast-food restaurants and convenience stores encourage overconsumption of unhealthy foods and consequently higher BMI, while the presence of supermarkets leads to increased intake of fruits and vegetables and thus lower BMI. Although associations between some types of food outlets and obesity have been reported, there have also been many null findings. Moreover, even when there is a positive association between food outlets and BMI, this association does not seem to be replicated in associations between distance to food outlets and diet (10).

Despite the widespread use of the term “neighborhood food environment,” there has been no consensus on what this term means in relation to geographic area. The White House Task Force on Childhood Obesity Report (3) references a single study, which was based on densities in postal and zip codes, for its recommendation to increase the number of supermarkets to reduce childhood obesity (11). However, a similar analysis using census tracts showed no such association (12). Although predefined administrative units may be more feasible for monitoring purposes, measures reflecting the actual distance to food outlets may identify disparities between communities more reliably because they show the real distance that people must travel to access food. Most studies were limited by grouping respondents into large geographic areas because there was no information on actual locations of respondents, so the sensitivity of results to distances between respondents and food outlets was not tested. However, there are a couple of exceptions for studies of adults. One study using data from California found that food outlets within walking distance (≤1.0 mile) were not strongly associated with dietary intake or BMI, although there were some significant associations beyond walking distance (13). Another study conducted longitudinally of young adults found no strong associations between supermarkets and diet quality and fruits and vegetables intake, but it found that fast-food consumption among low-income men was related to fast-food availability in areas beyond immediate walking distance (14).

The US Department of Agriculture created an interactive web-mapping tool to identify food deserts (15). In that tool, the Food Access Research Atlas, the criterion for a food desert is based on distance from residential address to a supermarket; a census tract is considered to have low access to food if a significant number or share of individuals in the tract is far from a supermarket. The original distance was 1 mile, but a second criterion was added using 0.5 miles to show how sensitive the food desert map is to the distance demarcation. The definition of physical access to food retailers is also evident in a publication by the Centers for Disease Control and Prevention that recommends that communities “improve geographic availability of supermarkets” and proposes the number of supermarkets in census tracts as a measure to assess a food environment (16).

This study analyzes the relationship between physical food outlets, diet measures, and BMI among adults in Los Angeles County and investigates whether results of associations are related to the definition of the geographic size of the food environment. It uses a standard administrative definition, a census tract, as well as buffers of varying sizes around respondents’ homes.

## Methods

Data from the 2011 Los Angeles County Health Survey (LACHS), a random-digit–dial telephone survey of the adult county population (aged 18 years or older, N = 8,036) were used (17). The LACHS is a health survey conducted periodically by the Los Angeles County Department of Public Health. The survey collects self-reported information on demographics, health behaviors, insurance status and access to health care, and health conditions. Data are weighted to be representative of the adult population of Los Angeles County. Participants were excluded from the analysis who were missing geographic data to determine distances to food outlets, who were missing data on height and weight to determine BMI, and who were missing data on food consumption indicators, resulting in an unweighted sample size of 5,185.

Measures related to food consumption were the self-reported number of sugar-sweetened beverages and number of servings of fruits and vegetables consumed per day and the frequency of fast-food consumption. Questions related to food consumption were, “How many total servings of fruits and vegetables did you eat yesterday?,” “On an average day, about how many sodas or sweetened drinks such as Gatorade, Red Bull, or Sunny Delight do you drink? Do not include diet sodas or sugar-free drinks. Please count a 12-ounce can, bottle, or glass as one drink,” and “How often do you eat any food, including meals and snacks, from a fast-food restaurant like McDonald’s, Taco Bell, Kentucky Fried Chicken, or another similar type of place?” (17). Each of these measures was standardized as weekly intake. Weight and height responses were used to calculate BMI (kg/m^2^). According to the definition of the World Health Organization (WHO), overweight was categorized as a BMI of 25 or more and obese as a BMI of 30 or more (18).

Five circular buffers of varying radii (0.25, 0.5, 1.0, 1.5, and 3.0 miles) were drawn and centered in each respondent’s residential address (or nearest cross street), and the number of food outlets by type was counted in each buffer (13,14,19). Straight line (Euclidean) distances were set using ArcMap 10.1 (ESRI) rather than actual travel distances using a road network. One-quarter mile or 400 meters is often considered a standard for walkability (eg, for transport planning), so the definition of the smallest area would include locations that were farther away using road networks. Food outlets, LACHS sample points, and buffers were overlayed to count the number of outlets by type lying inside those radii. The same analyses were also conducted using the census tract of the respondent’s residential address, instead of using circular buffers.

Food outlet data from the 2009 release of InfoUSA were used and classified into 5 types using the North American Industry Classification System (NAICS) (20): fast-food restaurants, convenience stores, small food stores, midsize grocery stores, and large supermarkets. Because no formal definition of fast food exists, this category included food outlets that offered pizza, burgers, tacos, and club sandwiches. The codes used for each category were a subsample of NAICS codes: 72221105–6 for fast-food outlets; 44512001 for convenience stores; and 44511001–3 for small food stores (annual sales of less than $1 million), grocery stores (annual sales of $1–$5 million), and supermarkets (annual sales of more than $5 million) (13).

Three types of regression models were used — negative binomial, ordinary least squares, and logistic — each to address a different type of association. The relationship between neighborhood food environment and dietary intake was analyzed through negative binomial models. Dietary intake measures (weekly consumption of fruits and vegetables, sugar-sweetened beverages, and fast food) were the dependent variables, and the numbers of food outlets by type (convenience stores, small food stores, midsize grocery stores, large supermarkets, and fast-food restaurants) in each radius were the explanatory variables. Different regressions were conducted for each dependent variable and for each buffer size. Average marginal effects (AMEs) and Bonferroni’s adjustment for multiple tests were also calculated. Models controlled for potential confounders with sociodemographic variables including sex, age, age squared, race/ethnicity (white, black, Hispanic, Asian/Pacific Islander, Native American, and other race or multirace), household size, educational level (not a high school graduate, high school graduate, some college, and college graduate or more), marital status (married or living together, single, and separated, divorced, or widowed), poverty level (income ≤100% of the federal poverty level [FPL]), and physical activity level (sedentary, some activity, and regular activity). Census 2010 data were used to control at the tract level for median annual household income, population density, and the ratio of white to nonwhite Latino population (21). All explanatory and control variables were divided by their standard deviation to homogenize units and their magnitudes.

The relationship between BMI and food environment was analyzed using ordinary least squares, where the dependent variable was BMI and food environment characteristics were the covariates. The same set of control variables at the individual and census tract levels previously described were used. Finally, the relationship between binary outcomes (overweight and obesity) and the food environment was analyzed using logistic regression models with the same set of controls previously described. In all analyses, separate regressions were conducted for each buffer size. This analysis resulted in 15 regressions and a total of 75 effects for each outcome: (5 buffer sizes) × (5 types of outlets) × (3 types of food or 3 measures of BMI). 

The density of food outlets per census tract was also analyzed by conducting the same models previously described, but instead of using number of outlets by buffer size we used number of outlets in census tract per 1,000 inhabitants and in census tract per square mile. Additionally, a sensitivity analysis was conducted including an additional measure of poverty level (income ≤200% of the FPL). This sensitivity analysis replicated all the models described but only for the survey respondents who were above and below 200% of the FPL. The statistical software used for all analyses was Stata version 12.1 IC (StataCorp LP). Models included sampling weights provided by LACHS. *P* values were estimated based on heteroskedasticity-robust standard errors from the Eicker–Huber–White sandwich estimator. Significance was set at the .05 level.

## Results

Of the approximately 10 million residents of Los Angeles County, 18% live below the poverty threshold. Forty-eight percent of the population is Hispanic/Latino; 29%, non-Hispanic White; 10%, African American; and 11%, Asian. The median household income is $56,000. The median census tract in Los Angeles County has an area of 0.45 square miles and 4,500 residents (21).

Adults in Los Angeles County consume on average 5.4 sugar-sweetened beverages each week, nearly 1 per day (Table 1). Survey respondents eat fast food on average once per week and eat 19.6 portions of fruit and vegetables per week. Average BMI was 27.5; 62.3% of survey respondents were overweight and 24.6% were obese.

**Table 1:** Characteristics of Respondents and Census Tracts, Los Angeles County Health Survey, 2011^a^

Characteristic	Value
**Dietary intake, mean no. of servings (IQR)**	
Sugar-sweetened beverages	5.4 (0–7)
Fast food	1.0 (0–2)
Fruits and vegetables	19.6 (14–28)
**Body mass index, kg/m^2^**	
Mean (SD)	27.5 (7.2)
Overweight category (≥25.0), no. (%)	3,057 (62.3)
Obese category (≥30.0), no. (%)	1,206 (24.6)
**Male, no. (%)**	2,572 (49.6)
**Mean age, (SD), y**	42.3 (16.8)
**Race/ethnicity, no. (%)**	
White, non-Hispanic	2,190 (42.2)
African American, non-Hispanic	472 (9.1)
Asian or Pacific Islander, non-Hispanic	684 (13.2)
Native American, non-Hispanic	148 (2.9)
Other race or multirace, non-Hispanic	156 (3.0)
Hispanic	1,535 (29.6)
**Mean no. of household members (IQR)**	3.6 (2–5)
**FPL ≤100%, no. (%)**	1,397 (26.9)
**Education, no. (%)**	
Not a high school graduate	1,271 (24.5)
High school graduate	1,205 (23.2)
Some college	1,455 (28.0)
College graduate or higher	1,254 (24.2)
**Marital Status, no. (%)**	
Married or living together	2,875 (55.9)
Divorced/separated/widowed	859 (16.7)
Single	1,413 (27.4)
**Physical activity level, no. (%)**	
Sedentary	565 (10.9)
Some activity	1,264 (24.4)
Regular activity	3,261 (62.9)
**Census tracts^b^**	
Population per square mile, mean (IQR), no.	13,525.4 (6,921–17,760)
Median household income, (SD), $	57,965.6 (27,155.7)
Mean non-Hispanic white (SD), %	50.3 (19.2)

Abbreviations: FPL, federal poverty level; IQR, interquartile range; LACHS, Los Angeles County Health Survey; SD, standard deviation.

a Percentages and means are weighted using LACHS sampling weights. Data are unweighted. Percentages may not sum to 100 because of rounding. Sample size was 5,185 people aged 18 years or older.

b Data source: Census Bureau, 2010 (21).

Number of outlets by type and radius size (data not shown) were nonoverlapping or mutually exclusive in radii of different size centered in the same residence. The largest number of food outlets in any buffer size was small food stores: 0.8 in a 0.25-mile radius, 57.8 within a 3.0-mile radius, and 3.9 within a census tract. The lowest number of food outlets in all buffers was midsize grocery stores: 0.04 within a 0.25-mile radius, 3.0 within a 3.0-mile radius, and 0.2 within a census tract. On average, in a radius of 0.5 miles of the respondent’s residence, there were 1.8 fast-food outlets, 0.5 convenience stores, 2.2 small food stores, 0.1 midsize grocery stores, and 0.7 supermarkets. There were more outlets of all types in areas of residents with lower income.

Only 8% of the effects of the food outlets on the dietary intake in all buffers (6 of 75) were significant. After applying Bonferroni’s adjustment, only 1.3% (1 of 75) were significant (Table 2). That effect corresponds to the number of fast-food restaurants in large, nonwalkable areas (3.0 miles) on the consumption of fast food. Most effects of the number of food outlets in all buffers (88%, or 66 of 75) on BMI were not significant (Table 3). After applying Bonferroni’s adjustment, only 1.3% (1 of 75) of the associations were significant; convenience stores within walkable distances (0.25 miles) was negatively associated to the probability of being obese. When analyzing the density of food outlets in census tract per square mile (data not shown), there was no significant effect after applying Bonferroni’s adjustment. Also, no significant effect was found between the density of food outlets in census tract per 1,000 population and the outcomes of interest.

**Table 2:** Estimated Change in Intake^a^ of Food Item, by Food Outlet Type^b^ and Food Environment^c^, Los Angeles County Health Survey, 2011

Food Outlet Type/Food Item	0.25 Miles		0.5 Miles		1.0 Miles		1.5 Miles		3.0 Miles	
	AME^d^	*P* Value^e^	AME^d^	*P* Value^e^	AME^d^	*P* Value^e^	AME^d^	*P* Value^e^	AME^d^	*P* Value^e^
**Fast-food restaurant**										
F&V	−0.070	.80	−0.795	.02	−0.252	.43	−0.223	.50	−0.860	.12
SSB	0.684	.15	0.969	.10	−0.815	.17	0.809	.31	0.050	.96
Fast food	−0.049	.07	0.036	.18	0.034	.24	0.037	.24	0.149^f^	.002
**Convenience Store**										
F&V	0.135	.60	0.469	.10	0.233	.55	0.191	.58	1.096	.05
SSB	−0.575	.27	0.029	.96	0.835	.22	0.112	.89	1.748	.12
Fast food	−0.029	.26	0.008	.75	−0.006	.84	0.060	.07	−0.074	.16
**Small food store**										
F&V	−0.149	.61	−0.312	.37	−0.216	.60	−0.198	.64	−0.773	.22
SSB	−0.200	.69	0.432	.53	0.993	.27	−0.310	.71	−1.204	.29
Fast food	0.035	.24	0.016	.63	0.025	.52	−0.007	.87	0.023	.68
**Midsize grocery store**										
F&V	0	.99	0.495	.07	0.020	.95	−0.192	.54	0.928	.11
SSB	0.044	.94	−0.742	.19	−0.663	.32	−0.743	.34	1.168	.25
Fast food	0.004	.85	0.008	.78	0.005	.87	−0.014	.66	0.007	.88
**Large supermarket**										
F&V	0.056	.84	−0.112	.73	0.214	.52	0.270	.52	−0.166	.75
SSB	−0.513	.38	−0.831	.17	−0.867	.21	−0.044	.96	−2.437	.04
Fast food	−0.006	.83	−0.013	.64	−0.062	.05	−0.093	.02	−0.129	.007

Abbreviation: AME, average marginal effect; F&V, fruits and vegetables; SSB, sugar-sweetened beverages.

a Number of times the item was consumed per week.

b Food outlet types are fast-food outlets, convenience stores, small food store, midsize grocery store, and large supermarkets.

c Food environment was defined by counting the number of food outlet types in each buffer of a certain radius (eg, 1.0 mile) centered on a respondent’s residence.

d AME measures an estimated change in the per-week frequency of consumption of each food item associated with 1 unit change in the regressor of interest. All regressors were divided by their standard deviations. Statistics were adjusted by using Los Angeles County Health Survey sampling weights.

e *P* values were calculated by using the *z* statistic obtained through negative binomial regression and based on standard errors estimated using the Eicker-Huber-White sandwich estimator.

f AME is significantly different from zero (at the 0.05 level) after applying Bonferroni’s adjustment for multiple comparisons. All 5 food outlet types were included in the regression models, and individual- and census tract–level characteristics were controlled for (but are not presented here).

**Table 3:** Estimated Change in BMI (kg/m^2^) and the Probability of Overweight or Obesity, by Food Outlet Type^a^ and Food Environment^b^, Los Angeles County Health Survey, 2011

Food Outlet Type/BMI	0.25 Miles		0.5 Miles		1.0 Miles		1.5 Miles		3.0 Miles	
	AME^c^	*P* Value^d^	AME^c^	*P* Value^d^	AME^c^	*P* Value^d^	AME^c^	*P* Value^d^	AME^c^	*P* Value^d^
**Fast-food restaurant**										
BMI	−0.035	.76	0.011	.87	0.049	.12	0.041	.15	0.019	.10
BMI ≥25.0 (overweight)	−0.007	.49	0.013	.21	0.015	.19	0.024	.04	0.034	.08
BMI ≥30.0 (obese)	−0.003	.76	0.000	.99	0.023	.02	0.014	.18	0.019	.26
**Convenience store**										
BMI	−0.516	.08	0.079	.64	−0.032	.74	−0.027	.73	−0.057	.12
BMI ≥25.0 (overweight)	−0.022	.03	0.001	.92	−0.009	.43	−0.002	.88	−0.020	.30
BMI ≥30.0 (obese)	−0.031^e^	<.001	−0.001	.88	−0.021	.04	0.011	.36	−0.039	.04
**Small food store**										
BMI	−0.133	.23	0.046	.43	−0.004	.84	−0.011	.44	−0.008	.11
BMI ≥25.0 (overweight)	−0.007	.51	−0.001	.94	−0.026	.09	−0.030	.07	−0.044	.05
BMI ≥30.0 (obese)	−0.015	.13	0.003	.82	0.011	.40	−0.005	.70	0.007	.71
**Midsize grocery store**										
BMI	0.353	.63	−0.162	.67	0.510	.02	−0.156	.35	0.110	.25
BMI ≥25.0 (overweight)	−0.004	.68	−0.009	.38	0.022	.07	−0.012	.32	0.018	.34
BMI ≥30.0 (obese)	0.006	.48	−0.003	.78	0.016	.14	−0.012	.24	0.001	.94
**Large supermarket**										
BMI	0.293	.39	−0.007	.96	−0.176	.01	−0.043	.49	−0.005	.83
BMI ≥25.0 (overweight)	−0.003	.73	−0.007	.53	−0.017	.16	−0.022	.11	−0.029	.12
BMI ≥30.0 (obese)	−0.006	.48	−0.001	.91	−0.024	.02	−0.012	.35	0.001	.94

Abbreviation: AME, average marginal effect; BMI, body mass index.

a Food outlet types are fast-food outlets, convenience stores, small food store, midsize grocery store, and large supermarkets.

b Food environment was defined by counting the number of food outlet types in each buffer of a certain radius (eg, 1.0 mile) centered on a respondent’s residence.

c AME on BMI is the estimated change in BMI (in kg/m^2^); AME on BMI ≥25.0 (or on BMI ≥30.0) is the estimated change in the probability of being overweight (or of being obese) associated with 1 unit change in the regressor of interest. All regressors were divided by their standard deviations. Statistics were adjusted by using Los Angeles County Health Survey sampling weights.

d *P* values for BMI were calculated by using the *t* statistic obtained through ordinary least squares regressions. *P* values for overweight (BMI ≥25.0) and obesity (BMI ≥30.0) were calculated by using the *z* statistic obtained through logistic regressions and based on standard errors estimated using the Eicker–Huber–White sandwich estimator.

e AME is significantly different from zero (at the 0.05 level) after applying Bonferroni’s adjustment for multiple comparisons. All 5 food outlet types were included in the regression models, and individual- and census tract–level characteristics were controlled for (but are not presented here).

In the sensitivity analysis (data not shown), there were significant associations only between the number of midsize grocery stores within 0.25 miles and the frequency of fast-food intake among respondents with an income above 200% of the FPL. No significant effect was found on food intake in respondents with income at or below 200% of the FPL. Significant effects of convenience stores in a 0.25-mile radius on the probability of being obese were found for respondents at an income at or below 200% of the FPL. These effects were similar to those of the models with the entire pool of respondents. There was no effect of the explanatory variables on BMI of respondents with an income above 200% of the FPL. All results held after conducting every model previously described without income, a variable potentially correlated with education.

## Discussion

Overall, no strong evidence emerged that local food environments affect diet or BMI of adults in Los Angeles County. There were few significant effects of 2 types of food outlets: fast-food outlets and convenience stores, but they represent only 1.3% (2 of 150) of all effects analyzed. Fast-food outlets within nonwalkable distances (3.0 miles) were positively associated with fast-food intake. The density of convenience stores within walkable distances (0.25 miles) was negatively associated with the probability of being obese, but it was not related to any other weight or dietary outcome. There was no association between the intake of fruits and vegetables or sugar-sweetened beverages and any type of food outlet in all buffers analyzed. Similarly, there was no association between BMI and fast-food outlets, small food stores, midsize grocery stores, or supermarkets.

This study does not provide evidence to support the hypothesis that the food environment within walkable distances affects BMI and diet of adults, as other studies do (19,22). Our analysis included the number of grocery stores and supermarkets present in the community, which is suggested as a community measure to prevent obesity (16), but it does not predict dietary and obesity outcomes.

This analysis suggests that the food environment within walkable distance in Los Angeles County is not a factor related to overweight, fruit and vegetable consumption, sugar-sweetened beverage consumption, or fast-food intake, which parallels the findings from other data sets (13,23,24). To the extent that there are significant associations, they seem to occur at distances beyond walking distance.

There are several limitations to this study. First, the data were cross-sectional. Second, because the data were self-reported, recall and social desirability may bias BMI (25), fast-food, sugar-sweetened beverage, and fruit and vegetable consumption. Food intake measures came from single questions for specific foods, not from a detailed food diary or a full 24-hour food recall. This single-item approach is standard for most surveys such as the Behavioral Risk Factor Surveillance System, California Health Interview Survey, and Early Childhood Longitudinal Survey, but it does not allow comparison of food quality measures. Third, complete geographic and indicator data were available for approximately 65% of the respondents. It is unclear whether respondents with missing information differed in any way from those included in the analysis. Fourth, the cooperation rate of the 2011 LACHS was 66% and, although comparable to other large state and national surveys of this type, reflects the ongoing challenges of conducting telephone-based surveys. Fifth, the food outlet data and survey dates were not identical. Business data listings are never complete or up-to-date. In previous work, we compared listings and on-the-ground verification in the City of Santa Monica and found excellent agreement for outlets belonging to chains, whether restaurants or retail, but not for small individually owned businesses. Field studies (26,27) found reasonably good predictive values but discrepancies at small scales. Furthermore, categorizing food outlets by type is insufficient to reflect outlet heterogeneity, and more detailed measures, such as ratings of food quality, could be more predictive for health outcomes. Finally, the buffer analysis used Euclidean distance rather than road networks, so even a small buffer (0.25 miles or 400 meters) may include outlets that residents consider to be outside walking distance.

This analysis focuses on the concept of access to particular types of stores. Access is a key element in the policy debate, as exemplified by the US Department of Agriculture’s Food Access Research Atlas, policy recommendations for supermarkets, or regulations such as the Los Angeles Fast-Food Ban, a zoning ordinance that prohibits opening a stand-alone fast-food restaurant in certain neighborhoods (28,29). However, store distance seems to consistently show little association with diet even when associations with BMI are reported (10,30).

The concept of neighborhood food environments has been the focus of the news media and policy makers, yet the evidence is not clear on whether promoting or discouraging a particular type of food outlet is an effective approach to promoting healthful dietary behaviors and a healthy weight. Initial findings in a new area of research — such as food environments — may be qualified over time, and both exact replication and conceptual replication of previous findings using alternative data sources and methods is a central theme for advancing scientific knowledge and informing policies. No single study completely addresses a research question, and this study can only contribute findings related to one aspect of the question. However, in Los Angeles County, the relationship between neighborhood food outlets and dietary intake or BMI is subtler than the relationship presented in the news media (8,9). The relationship appears to exist in larger geographic areas rather than within walking distances or in a typical urban census tract.

There are several reasons why the original idea that neighborhood outlets determine diet should be modified. The most obvious reason is that the importance of proximity has diminished in a highly motorized society, which may be more applicable to an area such as Los Angeles County than, for example, New York City. In addition to physical access to a certain type of food outlets, other dimensions that affect diet behavior are affordability, availability in those outlets, and cultural acceptability of the food.

## Figures and Tables

**Table:** 

**1. The activity supported the learning objectives.**				
**Strongly Disagree**				**Strongly Agree**
1	2	3	4	5
**2. The material was organized clearly for learning to occur.**				
**Strongly Disagree**				**Strongly Agree**
1	2	3	4	5
**3. The content learned from this activity will impact my practice.**				
**Strongly Disagree**				**Strongly Agree**
1	2	3	4	5
**4. The activity was presented objectively and free of commercial bias.**				
**Strongly Disagree**				**Strongly Agree**
1	2	3	4	5

## Post-Test Information

To obtain credit, you should first read the journal article. After reading the article, you should be able to answer the following, related, multiple-choice questions. To complete the questions (with a minimum 75% passing score) and earn continuing medical education (CME) credit, please go to http://www.medscape.org/journal/pcd. Credit cannot be obtained for tests completed on paper, although you may use the worksheet below to keep a record of your answers. You must be a registered user on Medscape.org. If you are not registered on Medscape.org, please click on the "Register" link on the right hand side of the website to register. Only one answer is correct for each question. Once you successfully answer all post-test questions you will be able to view and/or print your certificate. For questions regarding the content of this activity, contact the accredited provider, CME@medscape.net. For technical assistance, contact CME@webmd.net. American Medical Association's Physician's Recognition Award (AMA PRA) credits are accepted in the US as evidence of participation in CME activities. For further information on this award, please refer to http://www.ama-assn.org/ama/pub/about-ama/awards/ama-physicians-recognition-award.page. The AMA has determined that physicians not licensed in the US who participate in this CME activity are eligible for ***AMA PRA Category 1 Credits*** ™. Through agreements that the AMA has made with agencies in some countries, AMA PRA credit may be acceptable as evidence of participation in CME activities. If you are not licensed in the US, please complete the questions online, print the AMA PRA CME credit certificate and present it to your national medical association for review.

## Post-Test Questions

### Article Title: Neighborhood Food Environment, Diet, and Obesity Among Los Angeles County Adults, 2011

**CME Questions**

1. Which one of the following statements regarding the baseline data of participants in the current study is most accurate?
  - Participants consumed an average of 3 sugar-sweetened beverages (SSBs) per day
  - Participants consumed fast food about once per week
  - Participants consumed fewer than 5 servings of fruits and vegetables per week
  - Half the participants had a normal body mass index
2. Which one of the following was the most common food outlet, on average, within 0.5 miles of respondents' addresses in the current study?
  - Midsize grocery store
  - Small food store
  - Fast food outlet
  - Convenience store
3. Which one of the following relationships between food outlet distribution and consumption habits among participants in the current study was most significant?
  - Convenience stores within walking distance were associated with higher consumption of SSBs
  - Fast food outlets outside of walking distance were associated with higher consumption of fast food
  - Supermarkets within walking distance were associated with higher consumption of fruits and vegetables
  - Small food stores outside of walking distance were associated with lower consumption of SSBs
4. Which one of the following relationships between food outlet distribution and overweight/obesity among participants in the current study was most significant?
  - Fast food outlets within walking distance were associated with a higher risk for obesity
  - Convenience stores within walking distance were associated with a lower risk for obesity
  - Supermarkets within walking distance were associated with a lower risk for obesity
  - Small food stores outside of walking distance were associated with a higher risk for obesity

**Evaluation**
